# Summary of data reported to CDC's national automated biosurveillance system, 2008

**DOI:** 10.1186/1472-6947-10-30

**Published:** 2010-05-25

**Authors:** Jerome I Tokars, Roseanne English, Paul McMurray, Barry Rhodes

**Affiliations:** 1 The National Center for Public Health Informatics, Centers for Disease Control and Prevention, Atlanta, Georgia, USA

## Abstract

**Background:**

BioSense is the US national automated biosurveillance system. Data regarding chief complaints and diagnoses are automatically pre-processed into 11 broader syndromes (e.g., respiratory) and 78 narrower sub-syndromes (e.g., asthma). The objectives of this report are to present the types of illness and injury that can be studied using these data and the frequency of visits for the syndromes and sub-syndromes in the various data types; this information will facilitate use of the system and comparison with other systems.

**Methods:**

For each major data source, we summarized information on the facilities, timeliness, patient demographics, and rates of visits for each syndrome and sub-syndrome.

**Results:**

In 2008, the primary data sources were the 333 US Department of Defense, 770 US Veterans Affairs, and 532 civilian hospital emergency department facilities. Median times from patient visit to record receipt at CDC were 2.2 days, 2.0 days, and 4 hours for these sources respectively. Among sub-syndromes, we summarize mean 2008 visit rates in 45 infectious disease categories, 11 injury categories, 7 chronic disease categories, and 15 other categories.

**Conclusions:**

We present a systematic summary of data that is automatically available to public health departments for monitoring and responding to emergencies.

## Background

Automated surveillance systems are increasingly being adopted by public health agencies [1]. A 2007--2008 survey found that 83% of responding state, territorial, and large-city health departments in the United States had a syndromic surveillance system which tracks clinical data, and that two-thirds were either highly likely or somewhat likely to expand their use of this modality [2]. Syndromic surveillance systems are also maintained by the US Department of Defense (DoD) [3,4], the US Department of Veterans Affairs (VA), and by several countries other than the US [5-7]. BioSense, maintained by the Centers for Disease Control and Prevention (CDC), is the national automated biosurveillance system used in the US. This system is intended to improve the nation's capabilities for conducting real-time biosurveillance, and enabling health situational awareness through access to existing data from healthcare organizations across the country [8]. This system automatically receives and analyzes health data from a variety of sources and provides a secure web-based application that is available to federal, state, and local public health and hospital personnel [9,10].

BioSense was initially developed in 2003 as the early event detection component of the CDC Public Health Information Network (PHIN). The system began receiving national data feeds from VA and DoD outpatient clinics in 2003 and made analyses of these data available through a web-based application in 2004. In mid-2005, CDC began to emphasize the concept of situational awareness, i.e., the ability to monitor the number of ill persons during an outbreak over time and geographic area, regardless of the source of initial event detection. In late 2005, the program began to receive data from civilian hospitals. In some cases, the program has created a split feed, which sends hospital data simultaneously to CDC and to the applicable state health department. In early 2006, updated methods of data analysis and processing were developed and presented in a new version of the application [9]. During 2006--2008, data from several state and local automated surveillance systems began flowing to the system; currently, most civilian hospital data is received via this mechanism rather than from individual hospitals or hospital groups. Early attempts were made to recruit hospitals systematically in major population areas, but ultimately the facilities included in the system represent a convenience sample of hospitals, hospital systems, and state/local systems that are willing and able to supply appropriate electronic data. Thus, BioSense has been termed a system of systems.

The term "automated surveillance" includes a range of activities from syndromic to case-based surveillance. Syndromic surveillance, using primarily chief complaint and diagnosis data, provides the capability for simple all-hazards surveillance. In the context of automated surveillance, case-based surveillance refers to the use of more specific data types, such as microbiology laboratory reports, to implement fully electronic case-detection algorithms [11]. Electronic laboratory reporting, which automates delivery of laboratory reports but does not apply a case definition [12,13], might be considered an intermediate step between syndromic and case-based surveillance. The case-based approach is promising but requires substantial additional work before it can be widely practiced. Currently, information available in automated systems, including BioSense, consists mostly of syndromic data.

Analysis of chief complaint and diagnosis data requires that the records first be assigned to categories, often called syndromes. The various automated systems use diverse lists of these concepts[3,14,15]. BioSense-defined surveillance concepts include broader syndromes and more-specific sub-syndromes. The 11 syndromes (botulism-like, fever, gastrointestinal, hemorrhagic illness, localized cutaneous lesion, lymphadentitis, neurological, respiratory, rash, severe illness or death, specific infection) were developed in 2003 by a multi-agency working group to encompass the prodromes of infectious diseases potentially caused by bioterrorism [16]. In 2006, using information from the existing systems [3,14], 78 sub-syndromes were defined to permit analysis of more specific concepts (e.g., cough) and to encompass infectious diseases, injuries, chronic diseases, and ill-defined symptoms or exposures [9]. While limited information has been published on the frequency of syndrome visits [17-19], comprehensive information on this topic has not been presented. In this manuscript, we describe the current sources of data being reported to this national system and present rates of visits meeting syndrome and sub-syndrome definitions during 2008. These data will help users to understand the types of illness and injury that might be studied using the system, give users an idea of the frequency of visits for the syndromes and sub-syndromes in the various data types, and facilitate comparison with other systems.

## Methods

Data sources included in this analysis are DoD Military Treatment Facilities outpatient clinics; VA outpatient clinics; and civilian hospital outpatient clinics, emergency departments (EDs), and inpatient facilities (Table 1). All US DoD and VA facilities, but only some civilian facilities, provide data. The DoD data are primarily from outpatient clinics; however, approximately 15% of the visits were from patients seen in emergency facilities. These visits can not currently be differentiated in BioSense. From the DoD and VA clinics, only International Classification of Diseases, 9th Revision, Clinical Modification (ICD-9CM) coded final diagnoses are sent. Civilian facilities may send chief complaints, working (preliminary) diagnoses, and final diagnoses. Additional clinically rich data (i.e., microbiology laboratory, radiology text reports) are received from a subset of civilian hospitals but are not included in this report. All BioSense data come from existing databases used by the data sources for administrative or clinical purposes; no data are specifically entered into a database for this project.

**Table 1:** Primary data source, visit, and patient characteristics, BioSense, January-December 2008.

Data Source and Type	No. of facilities	Total visits, millions	Visits per facility per day, median*	Record receipt, median†‡	Age, median years‡	Gender, % female‡
DoD Outpatient Final Diagnosis	333	32.9	148, 34	2.2 days	29	43.5
VA Outpatient Final Diagnosis	770	54.4	48, 33	2.0 days	61	7.2
Civilian Hospitals Outpatient						
Chief Complaint	85	4.6	121, 12	0.3 hour	52	64.8
Working Diagnosis	62	1.8	44, 4	4.6 days	53	64.2
Final Diagnosis	72	2.7	64, 7	5.1 days	51	62.7
Civilian Hospitals ED						
Chief Complaint	532	18.1	76, 9	4 hours	35	55.8
Working Diagnosis	105	2.4	51, 55	1.8 days	35	56.1
Final Diagnosis	203	6.3	66, 70	5.1 days	35	56.4
Civilian Hospital Inpatient						
Chief Complaint	115	1.7	32, 18	4 hours	50	59.3
Working Diagnosis	78	0.8	22, 13	8.1 days	51	58.1
Final Diagnosis	81	1.1	27, 16	9.9 days	47	59.2

DoD denotes Department of Defense, VA denotes Veterans Administration, ED denotes emergency department.

* Values are for weekdays, weekends.

† Time from patient visit to receipt of data at CDC.

‡ For these columns, data is from December 2008.

All data sources submit limited demographic information (i.e., age, gender). While patient names and other identifiers are not received, for the VA and some civilian hospitals, a random number identifier (Patient ID) is included and permits linking data from an individual patient across multiple visits. The records for linking this Patient ID with identifiable patient data is stored in a database at the data source and can be accessed only by authorized personnel there. Incoming data are routinely monitored for completeness of data fields and consistency with the number of records routinely received from each data sources, and problems are corrected by working with the applicable data source; however, such checks can identify only large-scale problems and more detailed checking for data accuracy is beyond current capabilities.

ICD-9CM coded diagnoses are assigned to 1 or more of the 11 syndromes as per the 2003 working group document [16] and are separately assigned to sub-syndromes according to a reference table developed by CDC [9]. The same visit may be classified as showing >1 disease indicator; for example, a visit with a chief complaint of "asthma and shortness of breath" will be included in both the asthma and dyspnea categories. However, counts from these 2 categories are analyzed separately and not added together. Free-text chief complaints and diagnoses are first assigned to sub-syndromes and then the sub-syndromes assigned to syndromes. The free-text data are automatically parsed for specified keywords. For example, chief complaint keywords for the asthma sub-syndrome include "asthma," "bronchospasm," "chest tight," "reactive airway disease," "status asthmaticus," and misspellings and abbreviations for these terms. The reference tables, available from the authors, include: ICD-9CM diagnosis to syndrome, ICD-9CM diagnosis to sub-syndrome, free-text diagnosis to sub-syndrome, free-text chief complaint to sub-syndrome, and sub-syndrome to syndrome. The specific infection syndrome, which includes acute infection of known cause not covered in other syndrome groups, is used for diagnoses but not for chief complaints. A given diagnosis or chief complaint may be assigned to 1 or more syndromes and sub-syndromes. While visits may have >1 diagnosis listed, only some facilities send reliable data indicating which is the primary diagnosis; therefore, currently all diagnoses are assigned to syndromes and sub-syndromes, without an attempt to determine which is the primary reason for the visit.

For most analyses, data from January 1--December 31, 2008, are summarized in this report. Facilities submitting information on ≥1 visit during ≥50% of the days during this period are included. The number of visits meeting each syndrome and sub-syndrome definition was determined and the rates per 1000 total visits were tabulated. "Total visits" refers to the total number of patient visits and includes visits both assigned and not assigned to a syndrome/sub-syndrome. The rate per 1000 total visits is more informative than simple numerator count data (e.g., 10 visits for respiratory illness has a different meaning among 50 vs. 500 total visits), and is more appropriate than rates per total population, since the system does not capture visits from the total population. Age, gender, and time to record receipt were determined from data reported in December 2008. We determined the median time from each visit to receipt at CDC by subtracting the date and time of visit as reported by the data source from the date/time stamp assigned by CDC computers when the message was received at CDC.

## Results

During 2008, data were received from 333 DoD and 770 VA facilities (Table 1). Among civilian hospitals, ED chief complaint data was received from 532 hospitals and ED final diagnosis data from 203; data from outpatients and inpatients were received from a smaller number of facilities. Reported visits totaled 32.9 million for the DoD, 54.4 million for the VA, and 18.1 million for hospital ED chief complaints. The median number of visits per facility per weekday was 148 for the DoD, 48 for the VA, and 76 for hospital ED chief complaint. Markedly fewer visits occurred on the weekends for the DoD, VA, and civilian hospital outpatient clinics.

Median time to receipt of data at CDC was lowest for outpatient chief complaints (0.3 hour) and highest for hospital inpatient final diagnosis (9.9 days) (Table 1). For ED chief complaints, median time to receipt was 4 hours, but varied from 17 minutes if the data were sent directly from the hospital to CDC to 9.4 hours if the data were sent via a state or local system. Median age was lowest for DoD clinics (29 years) and highest for VA clinics (61 years). For most data sources, a majority of visits were made by females (e.g., ED chief complaint data 55.8% female), except for the DoD and VA clinics (43.5% and 7.2% female, respectively).

**Figure 1:**
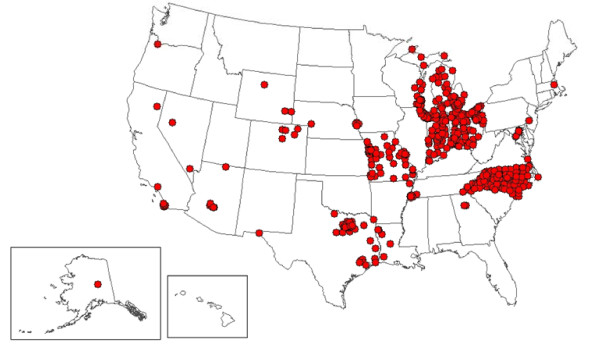
**Location of 532 civilian hospitals providing emergency department chief complaint data to BioSense, 2008**.

**Figure 2:**
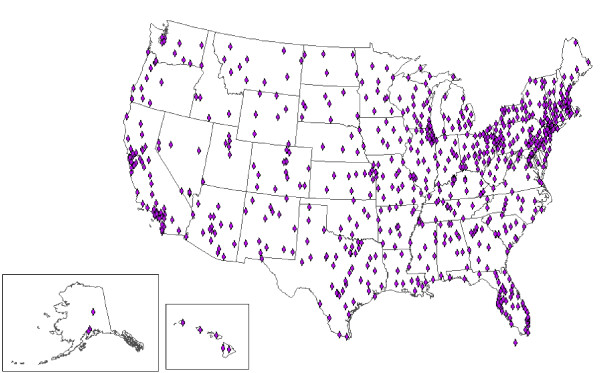
**Locations of 770 Veterans Affairs outpatient clinics providing data to BioSense, 2008**.

**Figure 3:**
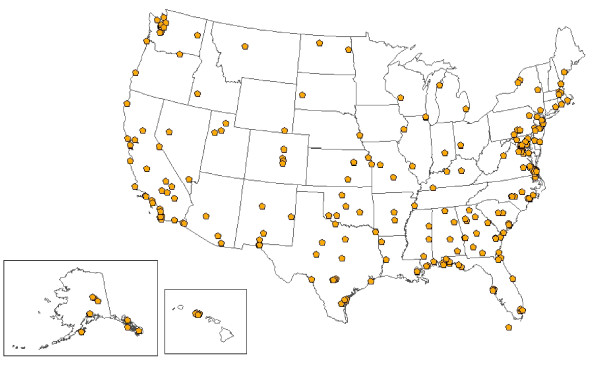
**Locations of 333 Department of Defense Military Treatment Facilities providing data to BioSense, 2008**.

At least 1 facility is located in each of the 50 states for the VA, in 47 states for the DoD, and in 25 states for civilian hospitals (Figures 1, 2 and 3). Civilian hospitals are clustered in certain states (Illinois, Indiana, Michigan, Missouri, North Carolina, Ohio, and Texas) having strong state or local systems that share data with BioSense. The number of VA and DoD facilities providing data has been similar each year, but the number of civilian hospitals providing data has increased from 331 in December 2006, to 534 in December 2007, and to 566 in December 2008 (data not shown).

Among the 11 syndromes, rates of ED chief complaint visits were highest for respiratory (205.6 per 1000) and gastrointestinal (144.0) and lowest for severe illness and death (0.3) (Table 2). These syndromes are also highest and lowest for other data sources. For a given syndrome, visit rates varied markedly among the data categories, e.g., the fever syndrome rate was highest for ED chief complaints (59.1) and lowest for VA final diagnosis (0.8). In part, this variability is due to differences in the reference tables used for chief complaints vs. diagnoses, the numbers of facilities reporting for each data type, and the numbers of chief complaints or diagnoses reported per visit. Lower rates for the civilian outpatient and VA may reflect the reality that many visits in these settings are for chronic disease follow-up rather than for acute disease treatment which would be reflected in the syndromes.

**Table 2:** Visit rates per 1000 total visits for 11 syndromes by data source, patient class, and data type, BioSense, January-December 2008.

Syndrome	ED CC	ED FD	IP CC	IP FD	OP CC	OP FD	DoD FD	VA FD
Botulism-like	2.9	7.2	3.9	17.3	4.5	3.4	1.4	5.2
Fever	59.1	47.6	27.9	36.9	7.1	5.8	7.8	0.8
Gastrointestinal	144.0	136.2	61.3	77.1	39.6	43.8	22.8	9.6
Hemorrhagic illness	25.1	15.7	26.8	41.7	10.0	6.6	2.9	5.7
Localized cutaneous lesion	23.4	30.6	19.1	35.7	4.0	9.0	6.2	6.5
Lymphadenitis	0.5	3.9	0.6	4.8	0.4	3.3	1.4	0.6
Neurological	53.7	53.2	27.1	35.9	11.6	14.2	7.8	8.1
Respiratory	205.6	233.8	129.1	199.2	62.9	80.2	73.0	26.9
Rash	19.9	14.9	0.8	8.4	2.5	5.3	9.7	5.8
Severe illness or death	0.3	2.8	2.1	3.2	0.7	0.2	0.0	0.0
Specific infection	--*	19.4	--*	31.6	--*	4.1	6.5	3.0

Abbreviations: CC, chief complaint; ED, hospital emergency department; FD, final diagnosis; DoD, Department of Defense; IP, hospital inpatient; OP, hospital outpatient; VA, Veterans Affairs.

*Chief complaints are not included in the specific infection syndrome.

Of the 78 sub-syndromes, 45 are classified under the infectious disease syndromes (Table 3). Fourteen sub-syndromes are classified under the respiratory syndrome, 7 under the neurological syndrome, and smaller numbers under other syndromes. Among ED chief complaint visits, the highest rates were for abdominal pain (82.9 per 1000), chest pain (72.0), and nausea and vomiting (65.3). Among ED final diagnosis visits, the highest rates were for abdominal pain (81.4), chest pain (63.5), and upper respiratory infection (59.1). As expected, sub-syndromes that capture symptom concepts had higher rates for chief complaints than for diagnoses; e.g., for ED data, the dyspnea (shortness of breath) rate was 52.2 for chief complaints vs. 28.4 for diagnoses. The converse was true for sub-syndromes that capture diagnostic concepts; e.g., the asthma rate is 46.5 for diagnoses vs. 12.7 for chief complaints. Sub-syndromes reflecting more severe disease had higher rates in inpatients: e.g., the diagnosis rate for pneumonia was 47.3 for inpatients, 19.3 for ED patients, and 4.0 for outpatients.

**Table 3:** Sub-syndromes associated with infectious disease syndromes: visit rates per 1000 total visits by data source, patient class, and data type, January-December 2008.

Syndrome Sub-syndrome	ED CC	ED FD	IP CC	IP FD	OP CC	OP FD	DoD FD	VA FD
Botulism-like								
Dysphagia	0.9	1.5	1.5	13.6	3.8	3.7	0.9	1.9
Paralysis	1.2	2.0	1.6	10.0	0.5	1.1	0.3	3.0
Speech disturbance	0.9	1.8	0.9	6.7	0.3	0.7	0.2	0.7
Fever								
Fever	58.2	31.5	18.4	14.9	6.8	4.9	3.7	0.6
Septicemia	0.6	5.4	10.6	28.5	0.3	0.6	0.5	0.2
Viral infection, unspecified	1.6	16.4	0.3	4.7	0.1	1.5	4.2	0.2
Gastrointestinal								
Abdominal pain	82.9	81.4	36.7	23.4	27.3	29.0	9.0	4.5
Anorexia	1.4	1.6	0.4	3.5	0.2	0.3	0.2	0.2
Diarrhea	16.1	18.6	5.8	14.0	4.4	5.2	3.3	2.0
Food poisoning	0.3	0.2	0.1	0.4	0.0	0.0	0.1	0.0
Intestinal infection	1.0	1.9	5.1	0.9	0.6	0.3	0.1	0.1
Nausea and vomiting	65.3	54.3	19.2	18.4	6.0	5.9	4.8	1.1
Hemorrhagic illness								
Coagulation defects	0.1	1.4	1.5	6.4	0.1	2.1	0.2	2.3
Gastrointestinal hemorrhage	5.9	5.5	11.1	10.7	1.4	2.4	1.5	1.8
Hemorrhage	21.7	1.8	22.6	1.9	9.3	0.1	0.0	0.1
Purpura and petechia	0.1	2.7	0.2	19.6	0.1	2.1	0.6	1.1
Localized cutaneous lesion								
Insect bites	6.0	3.6	0.1	1.2	0.4	0.3	1.0	0.3
Skin infection	17.9	31.6	19.1	31.1	3.7	5.8	7.0	4.6
Lymphadenitis								
Lymphadenopathy	0.5	2.8	0.6	4.1	0.4	3.0	1.1	0.6
Neurological								
Alteration of consciousness	9.9	4.0	11.7	6.1	2.1	0.7	0.3	0.2
CNS inflammatory disease	0.3	1.8	1.2	2.2	0.3	0.3	0.2	0.1
Convulsions	9.9	10.5	8.4	10.7	2.6	4.2	0.5	3.1
Gait abnormality	0.9	1.4	0.2	7.7	0.4	2.3	1.0	2.9
Headache	33.2	39.1	6.5	13.9	6.4	9.0	6.4	4.5
Meningismus	0.6	0.0	0.1	0.1	0.1	0.0	0.0	0.0
Photophobia	0.3	1.9	0.0	0.2	0.0	0.0	0.1	0.2
Respiratory								
Asthma	12.7	46.5	7.9	60.0	5.1	15.3	8.9	5.7
Bronchitis and bronchiolitis	1.6	26.7	4.2	14.5	1.5	4.0	6.7	1.6
Chest pain	72.0	63.5	51.0	37.8	16.6	17.2	5.9	7.2
Cough	38.3	27.1	2.9	5.5	13.7	12.9	4.7	3.1
Cyanosis and hypoxemia	0.7	3.5	6.5	19.9	0.5	0.9	0.3	0.5
Dyspnea	52.2	28.4	34.7	15.0	13.6	16.8	4.6	5.5
Hemoptysis	1.5	1.2	0.9	2.9	0.3	0.5	0.1	0.3
Influenza-like illness	5.9	4.9	0.8	2.0	0.5	0.4	0.5	0.2
Otitis media	16.2	21.9	0.8	6.9	2.4	4.4	10.1	1.3
Pleurisy	0.5	4.2	2.4	18.1	1.0	2.7	0.4	0.6
Pneumonia	3.7	19.3	26.6	47.3	3.8	4.0	3.6	0.1
Respiratory failure	0.3	8.0	8.7	35.8	0.1	8.2	0.5	1.3
Respiratory syncytial virus	0.3	0.9	1.4	2.6	0.1	0.2	0.2	0.0
Upper respiratory infection	36.5	59.1	2.9	17.9	9.6	11.7	35.5	4.4
Rash								
Rash	19.9	9.5	0.8	3.4	2.5	2.9	2.4	2.1
Severe illness or death								
Coma	0.2	0.2	0.7	1.2	0.8	0.0	0.0	0.0
Death	0.3	0.5	0.8	0.1	0.1	0.0	0.0	0.0
Shock	0.1	1.3	0.8	9.8	0.1	0.1	0.1	0.0
Specific infection								
Specific infection	--	19.4	--	31.6	--	4.1	6.5	3.0

Abbreviations: CC, chief complaint; ED, hospital emergency department; FD, final diagnosis; DoD, Department of Defense; IP, hospital inpatient; OP, hospital outpatient; VA, Veterans Affairs.

Thirty-three of the sub-syndromes do not fall under a syndrome category, including 11 injury concepts, 7 chronic disease concepts, and 15 other concepts (Table 4). Among the injury sub-syndromes, the most commonly reported ED chief complaints were injury not otherwise specified (rate 71.0 per 1000 visits), falls (36.5), and open wounds (32.1); the most common diagnoses were sprains and strains (58.5), falls (57.7), and fractures and dislocations (37.1). Among the chronic disease concepts, hospital inpatient diagnoses had the highest rates, with hypertension, diabetes mellitus, and ischemic heart disease most frequent; however, such concepts are generally not the primary reason for the visit. Among the 15 other concepts, the most common ED chief complaints were dizziness (rate 19.7 per 1000 visits), malaise and fatigue (19.0), and edema (16.4).

**Table 4:** Sub-syndromes not associated with syndromes: visit rates per 1000 total visits, by data source, patient class, and data type, January-December 2008.

Category Sub-syndrome	ED CC	ED FD	IP CC	IP FD	OP CC	OP FD	DoD FD	VA FD
Injury								
Bites, animal	6.8	6.6	0.5	2.0	0.6	0.5	0.7	0.2
Burns	3.2	3.7	1.1	1.8	0.7	0.8	0.9	0.3
Carbon monoxide poisoning	0.2	0.2	0.1	0.1	0.0	0.1	0.0	0.0
Falls	36.5	57.7	5.9	30.4	2.3	5.3	3.0	1.3
Fractures and dislocations	4.4	37.1	22.7	30.8	7.7	14.5	10.5	5.0
Heat, excessive	0.3	0.6	0.1	0.3	0.0	0.1	0.2	0.0
Injury, not otherwise specified	71.0	30.2	8.1	4.3	7.4	4.4	1.5	2.0
Motor vehicle traffic	22.7	28.9	2.7	9.0	1.0	1.3	1.0	0.2
Open wound	32.1	25.3	7.1	8.4	1.2	1.8	1.7	0.7
Poisoning by medicine	4.0	4.8	2.5	6.5	0.2	0.4	0.3	0.1
Sprains and strains	2.4	58.5	1.0	9.4	2.2	7.7	22.0	4.0
Chronic diseases								
Cardiac dysrhythmias	5.0	32.4	8.8	118.3	7.8	22.5	6.9	30.7
Cerebrovascular disease	3.9	10.8	18.1	43.3	3.7	9.8	1.6	6.8
COPD	1.5	10.9	12.1	27.2	3.4	2.5	0.6	2.3
Diabetes mellitus	5.7	68.5	14.7	144.1	9.2	65.1	17.8	92.9
Heart disease, ischemia	2.2	36.2	24.4	132.5	8.7	21.6	3.7	36.4
Hypertension	8.5	128.4	14.8	244.5	34.0	96.8	31.8	124.4
Neoplasms	1.4	26.8	33.1	95.8	30.0	78.5	18.7	35.0
Other								
Anemia	1.0	24.6	12.9	143.7	7.7	26.7	5.7	15.9
Dehydration	2.9	20.2	14.8	54.2	0.8	2.6	1.3	0.6
Dizziness	19.7	16.0	5.0	6.9	3.7	5.3	2.4	3.4
Edema	16.4	4.7	3.8	7.0	6.9	4.7	0.8	4.2
Hypotension	1.3	5.4	4.8	29.7	2.1	0.8	0.4	1.3
Jaundice	0.3	0.5	1.3	1.9	1.6	0.4	0.1	0.2
Malaise and fatigue	19.0	13.6	1.7	10.7	5.2	11.1	2.3	1.8
Mental disorders	6.2	168.2	1.2	236.9	0.5	49.0	80.7	211.3
Migraine	5.8	13.1	0.5	11.3	0.7	2.2	4.8	2.9
Myalgia	4.1	9.7	0.3	7.7	0.7	2.9	3.0	2.4
Numbness	9.0	8.2	2.1	4.8	2.1	3.3	1.8	1.8
Pregnancy, childbirth	11.5	25.0	89.5	98.9	17.4	27.0	15.1	0.1
Syncope and collapse	12.0	12.6	13.3	12.7	2.5	3.0	1.5	1.4
Urinary tract infect	6.3	43.8	14.2	62.6	6.9	13.1	6.4	3.0
Visual impairment	2.2	1.3	0.6	5.1	0.9	0.6	0.2	4.1

Abbreviations: CC, chief complaint; ED, hospital emergency department; FD, final diagnosis; DoD, Department of Defense; IP, hospital inpatient; OP, hospital outpatient; VA, Veterans Affairs.

## Discussion

BioSense is a system-of-systems, with data being received from federal partners, existing health department systems, hospital systems, and individual hospitals. In 2008, the primary data sources were 532 civilian hospital EDs, 333 DoD facilities, and 770 VA facilities. These data sources vary markedly in their geographic coverage, population coverage, demographics, and timeliness of reporting. Demographically, visits to BioSense EDs are reasonably representative of the U.S ED population [20,21]; DoD visits, which are made by active duty personnel, retired persons, and family members, represent a younger population with a higher proportion of males; and VA visits represent an older and heavily male population. Since 2006, most of the growth in the system has been through receipt of data from existing state or local health department systems that provide ED chief complaint data. Time from patient visit to data receipt at CDC was shortest for civilian hospital chief complaint data, especially if the data was sent directly from the hospital rather than via a state or local system. A detailed comparison of ED data from BioSense with a nationally representative survey performed by the National Center for Health Statistics is underway [20,21]. However, the 2 most common reasons for visit (abdominal pain and chest pain) are the same in both systems. We estimate that in 2008 BioSense captured chief complaints from about 14--15% of all US ED visits.

We have summarized visit rates for both the broader syndromes and narrower sub-syndromes. Because they capture more specific concepts, sub-syndromes may be more useful. For example, during a 3-day period of smoke exposure due to wildfires in San Diego in October 2007, ED chief complaint visits increased 22% above the previous 28 days for the respiratory syndrome; in comparison, increases were larger for 2 respiratory sub-syndromes, dyspnea (50%) and asthma (182%) [22]. Automated surveillance was originally geared to infectious diseases, and many of the sub-syndromes fall under infectious categories such as respiratory and gastrointestinal. However, we also include sub-syndromes related to injuries, chronic diseases, and a number of general concepts not fitting in any of the above categories (i.e., malaise and fatigue). The breadth of concepts that can be monitored, albeit in a simple manner, is a strength of this system.

At least 3 methods are commonly used to make syndrome assignments from chief complaint data. The Real-Time Outbreak Detection System (RODS), which uses 7 syndromes, employs a training dataset of ED records which have been assigned manually to syndromes by experts; this dataset then is used to train CoCo, a naïve Bayesian classifier, to make the free-text chief-complaint-to-syndrome assignment during production data analysis [15]. The Early Notification of Community-Based Epidemics (ESSENCE) system, which uses variable numbers of syndromes and sub-syndromes in different versions, first normalizes the text to remove punctuation and expand abbreviations and then assigns the normalized text to a concept using human-assigned weights [3]. Other systems, including the Early Aberration Reporting System (EARS) [14] and the New York City system [18], use a text-parsing method, similar to that used by BioSense, which scans for keywords, misspellings, and abbreviations and then makes the category assignments.

In comparing BioSense with other commonly-used automated surveillance systems such as EARS, ESSENCE, or RODS, it is useful to distinguish between the BioSense program, which supports a number of activities including collation of numerous data types from around the U.S., vs. the BioSense application, which displays data and analyses. Other systems do not have a function comparable to the BioSense program, but instead provide software tools for a locality to analyze its own data. In comparison with the BioSense application, other systems use different surveillance concepts (as noted above), different statistical methods for finding data anomalies, and different interfaces for displaying results. An evaluation of the sensitivity and specificity of statistical methods used in BioSense has been published [19]; this includes a comparison with methods used in EARS but not with those used in other systems. A study funded by BioSense found that, because of greater familiarity and engagement, epidemiologists generally preferred to use systems that they developed rather than the BioSense application [23]; these results are useful as plans, outlined in the Conclusion, are being made to revise the BioSense program.

The BioSense program includes a number of activities not otherwise described in this manuscript. The BioIntelligence Center, composed of several analysts, reviews the data daily and provides reports to state and local health departments and to the CDC Emergency Operations Center. A specialized Influenza Module [24,25] summarizes data from 3 traditional sources supplied by the Influenza Division at CDC [26], and from 5 automated sources via BioSense. The sub-syndrome designated "influenza-like illness" captures free-text data that mention "flu" or "influenza" and ICD-9CM codes of 487; however, the Influenza Module uses the following combination of sub-syndromes for influenza surveillance: influenza-like illness or (fever and cough) or (fever and upper respiratory infection). Computer infrastructure installed by CDC is used to forward notifiable disease laboratory data to state health departments from 40 hospitals and 1 national laboratory. Plans to enable messaging from a second national laboratory are in progress. In addition, funding has also been provided to support original research, evaluation of syndromic surveillance and BioSense [23], and awards to the Centers of Excellence in Public Health Informatics [27].

Evaluation of the validity of the data received by automated systems is difficult, but a number of points support the validity of BioSense data. When new data sources are added, a one-week sample of data are scanned by technical personnel for data quality problems and adherence to data dictionary standards; thereafter, if the typical number of incoming records changes, corrective action is taken. As part of a BioSense-funded cooperative agreement, a validation of >9,000 records from two North Carolina hospitals showed >99% agreement with data received by CDC [28]. Biologically plausible increases in visits have been found for asthma associated with a wildfire [22], falls associated with winter weather [29], and burns associated with Independence Day [30]. Seasonal trends in influenza-like illness [25], heat injury [31], asthma [32], and gastrointestinal disease [33] follow expected patterns. Finally, a number of 1-day increases in visits at single hospitals have been linked via newspaper reports or personal communications to known incidents [34]; on three occasions, such increases were due to artificial records introduced into hospital ED systems during preparedness drills (unpublished information).

Limitations of this study include representation of only a convenience sample of civilian hospitals and an inability to perform detailed comparisons of visits at individual facilities with data received by the system. The rates per 1000 total visits presented represent proportional morbidity at facilities providing data rather than population-based incidence. Caution should be used in comparing these rates among the data sources because of differences in the numbers of facilities reporting, varying numbers of chief complaints or diagnoses provided per visit, and the use of different reference tables used to assign chief complaints vs. diagnoses to syndromes and sub-syndromes. There is potential misclassification because of limitations of patient-reported chief complaints, which are subjective, and diagnosis codes, which have well-recognized limitations. Additionally, the same patient may contribute >1 visit on different days, and follow-up visits for recheck may be particularly high in systems such as the DoD where patients do not have to pay for such visits. While the same visit may be classified as showing >1 disease indicator, counts from these 2 categories are analyzed separately and not added together. BioSense, like other automated systems, can monitor seasonal influenza activity [24,25] and recognize large increases in visits for some general surveillance concepts; however, a more substantial contribution to public health practice awaits the ability to access data that is more specific than chief complaints and diagnoses. Nevertheless, to our knowledge this report presents the largest collation of automated surveillance data yet assembled.

During its first 6 years of operation, the system has had a number of problems, most of which have been corrected or will be addressed in the near future. First, certain key variables, e.g., diagnosis priority at hospital facilities, and clinic type and patient identifier at DoD facilities, either were not available or were inadvertently excluded. During 2003--2006, the application displayed sentinel alerts based on ICD-9CM coded diagnoses from the VA and DoD systems, in some cases due to miscodes at the facility such as "plaque" being coded as "plague." Current processes avoid this problem. During 2005--2006, program insistence on receipt of data directly from hospitals was expensive and created resentment among some state and local health departments; the current approach emphasizing access to data through existing state or local systems or Health Information Exchanges is more fruitful. A number of problems have been identified in chief complaints and diagnosis mapping tables, e.g., "sore throat" being assigned to the localized cutaneous lesion syndrome because of the word "sore." The BioSense application has expanded functionality but lacks key features such as the ability to perform free-text searches or to create custom syndromes using terms such as "fever and cough." The capability to share datasets with health departments and research partners is hampered by data use agreements as well as technical issues. Finally, procedures for data receipt, warehousing, and pre-processing have not been flexible enough to allow revisions to be made quickly.

## Conclusions

We present the first systematic summary of data that is currently being received by the CDC BioSense program. This data is automatically processed and made available to public health departments for monitoring and responding to emergencies. This summary should help users and policy-makers understand the current uses and limitations of the data, and contribute to efforts to improve the field of automated surveillance. BioSense is being extensively revised to advance nation-wide real time biosurveillance capability and capacity. Principles will include continued expansion of biosurveillance capacity through fostering state and local systems, replacement of the national datasets currently maintained by CDC with a series of jurisdiction-specific datasets created and maintained by state and local systems (federated data model), continued promotion of electronic laboratory reporting, and increasing use of inexpensive, light-weight, software tools. In upcoming years, these initiatives will continue to expand the scope and utility of automated surveillance systems.

## Abbreviations

CC, chief complaints; CDC, Centers for Disease Control and Prevention; COPD, Chronic obstructive pulmonary disease; DoD, Department of Defense; ED, hospital emergency department; FD, final diagnosis; ICD-9CM, International Classification of Diseases, 9th Revision, Clinical Modification; IP, hospital inpatient; OP, hospital outpatient; PHIN, Public Health Information Network; VA, Veterans Affairs

## Competing interests

The authors declare that they have no competing interests.

## Authors' contributions

All authors read and approved the final manuscript. JT planned the project, designed the data tables, and drafted the manuscript. RE designed the analytic datasets, analyzed data, and edited the manuscript. PM created datasets for analysis and assisted with data analysis. BR provided overall guidance for the project and assisted with manuscript preparation

## Pre-publication history

The pre-publication history for this paper can be accessed here:

http://www.biomedcentral.com/1472-6947/10/30/prepub
